# Transient self-organisation of DNA coated colloids directed by enzymatic reactions

**DOI:** 10.1038/s41598-019-43720-7

**Published:** 2019-05-14

**Authors:** H. Dehne, A. Reitenbach, A. R. Bausch

**Affiliations:** Lehrstuhl für Zellbiophysik, Technische Universität München, James-Franck-Str. 1, D-85748 Garching, Germany

**Keywords:** Physical chemistry, Biomaterials, Biological physics

## Abstract

Dynamic self-organisation far from equilibrium is a key concept towards building autonomously acting materials. Here, we report the coupling of an antagonistic enzymatic reaction of RNA polymerisation and degradation to the aggregation of micron sized DNA coated colloids into fractal structures. A transient colloidal aggregation process is controlled by competing reactions of RNA synthesis of linker strands by a RNA polymerase and their degradation by a ribonuclease. By limiting the energy supply (NTP) of the enzymatic reactions, colloidal clusters form and subsequently disintegrate without the need of external stimuli. Here, the autonomous colloidal aggregation and disintegration can be modulated in terms of lifetime and cluster size. By restricting the enzyme activity locally, a directed spatial propagation of a colloidal aggregation and disintegration front is realised.

## Introduction

Self-assembly of colloidal particles is a powerful concept to build functional materials^1–10^. The resulting properties can be tuned by the choice of the single building blocks and their interactions^11–17^. DNA coated colloids have proven to be well suitable, as the programmability and specificity of their interactions allow a fine control of the formation of a diverse set of structures^18–23^. At high interaction energies amorphous gel like structures can be tightly controlled by fine tuning the kinetic pathways leading to the dynamic arrest^24–27^. In multicomponent systems, the interactions can be activated at different stages, which leads to the formation of scaffolds, able to guide the subsequent aggregation processes and thereby to the formation of higher hierarchical structures^28^. The DNA functionalised colloidal structures realized until now have in common that the finally formed structures are stable and static in time.

One approach to control the time course of colloidal aggregation is the use of external stimuli, which has been realized by controlling phosphorylation/dephosphorylation reactions^29^ or temperature shifts^30,31^. The dynamic transition from colloidal aggregation to disintegration in time without external intervention, requires a precise control of the underlying reaction kinetics. Such transient and autonomous control of colloidal aggregation has been realized by tuning the hydrophobicity of single building blocks with a simultaneous limited energy supply^32^. In this promising approach, the transient switch between colloidal aggregation and disintegration was achieved without external stimuli, yet lacks the selectivity of DNA hybridisation. For polymeric gels external control and autonomous structure formation processes have already been shown to be a promising route for the formation of active materials with unique properties^33–35^. The coupling of the selectivity and the dynamic control of DNA hybridisation of colloidal interactions would open up a new set of autonomously responding material classes, with the potential of hierarchical structure formation starting from the micron-scale.

**Figure 1:**
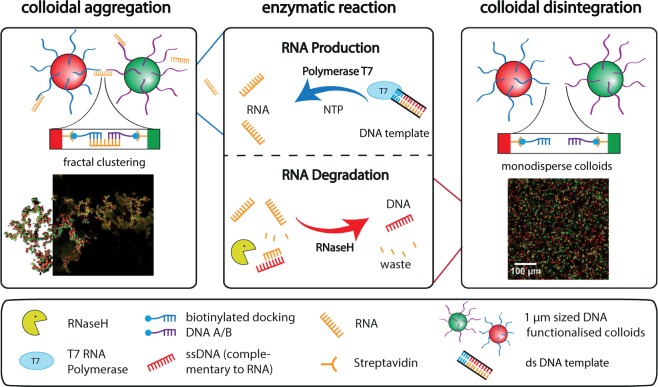
Colloidal structure formation coupled to enzymatic reactions. 1 μm sized polystyrene colloids are functionalized with two sorts of ssDNA docking strands and can be distinguished via fluorescence. The structure formation is induced by complementary RNA-linker strands which link the two different species of colloids. The production of the RNA-linker is realised using a T7 Polymerase under use of free dsDNA templates and NTPs in solution. The disintegration process of the so formed colloidal clusters is achieved by the activity of the ribonuclease *RNaseH*, which degrades the RNA-linkers once bound to DNA.

Here, we present the realization of an autonomous transient colloidal aggregation of DNA coated colloids driven by competing enzymatic reactions (Fig. 1). The enzyme *T7 Polymerase* is used to synthesize RNA-linker strands to induce the specific aggregation of micron sized colloidal particles (S1). A double-stranded DNA sequence is used as a template strand for the RNA-linker and the reaction is driven by NTP. The subsequent disintegration of the colloids is realised by the presence of the ribonuclease *RNaseH*. It degrades the RNA-linker strands once hybridized with DNA. By tuning the enzymatic kinetics, we can control the transient nature of the aggregation and subsequent disintegration process without external intervention during the structure formation process. The modulation of local enzyme activity leads to a spatiotemporal control of the autonomous transient structure formation process.

## Results

### Colloidal aggregation

**Figure 2:**
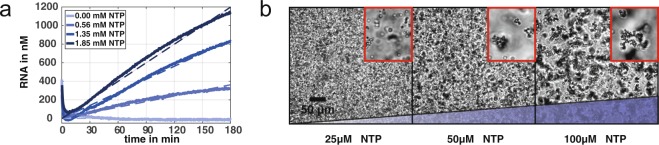
Colloidal aggregation induced by RNA production. (**a**) The RNA production was measured over time. Here, the T7 polymerase and the DNA template concentrations were kept constant while the fuel NTP was varied. A linear production rate in the nanomolar range can be observed over three hours of polymerisation. The dashed lines represent linear fits, which obey a linear dependency. The RNA concentration is visualised by the DNA/RNA intercalator SyGr II and can be converted into the effective RNA concentration (S2). The decrease of the signal in the beginning is due to the heating of the chamber, because the dye is more fluorescent at room temperature. (**b**) The NTP dependent RNA production is transferred to the colloids and monitored using bright field microscopy. The structure formation is depicted after one hour, showing that the enzymatic reaction of the RNA linker strands indeed induces the colloidal aggregation. The average cluster size was determined in a.U. using image analysis and rises with the NTP concentration (26, 53 and 112 a.U., respectively).

As a first step, the colloidal aggregation was coupled to the enzymatic reaction. Therefore, we monitored the production of RNA-linker using fluorescence spectroscopy (Fig. 2a). A constant production rate of the RNA-linker can be measured over three hours of polymerisation. The production speed can be tuned by varying the NTP concentration and increases linearly with the NTP concentration (S3). In a next step, we added the binary mixture of DNA functionalized colloids to solutions with different NTP dependent RNA productions. We imaged the colloidal structure formation after one hour of RNA polymerisation (Fig. 2b). The size of the colloidal aggregates increases successively with the NTP concentration, as a result of the RNA production speed. At low concentration of NTP (25 µM) the aggregation speed is slowed down and the arising clusters consist of just a few colloids. In contrast, high NTP concentrations (100 µM) result in colloidal clusters with the size of several 100 μm. Thus, we managed to control the speed of colloidal aggregation, by tuning the enzymatic production of RNA-linker. Additional experiments were performed for different NTP concentrations, proving the control of colloidal aggregation speed by measuring the cluster size over time (S4).

It is important to mention, that the control of speed can only be achieved for production rates below a certain production rate (~10 nM/min). Above this speed, the polymerase produces a sufficient amount of RNA (>50 nM) to enable optimal aggregation conditions in just a few minutes. Here, the speed of the aggregation process is only limited by the diffusion of the colloids and further increase of RNA-linker does not result in enhanced aggregation speed. Additionally, we used confocal microscopy to prove the selectivity of the RNA-linker caused by the DNA/RNA hybridisation (S5). Aggregation can only be observed, when both species of the DNA functionalised colloids are present. Since the different colloidal species can be distinguished by their fluorescence, the binding of colloidal species among themselves can be excluded.

**Figure 3:**
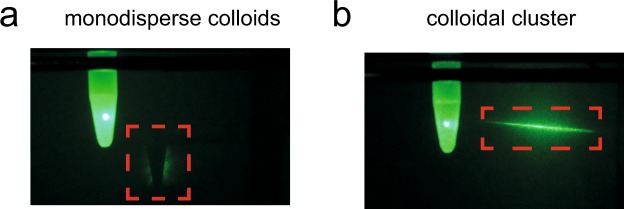
Optical properties of the colloidal solution. (**a**) Colloids are illuminated by a green 1 mW laser beam. The sample containing monodispersed colloids absorbs the laser beam, which can be seen by the outline of the sample holder (red dotted line) on the detection wall. The colloidal volume fraction is too high and causes absorption and scattering of the light beam. (**b**) In contrast, the colloidal gelation leads to the transmission of the laser beam. Here, huge volume fractions arise, where no colloids are present.

Finally, we show that the aggregation of the colloids is also able to tune optical properties of the solution. Therefore, we illuminated a sample with a green laser before and after the enzymatic induced clustering. The monodisperse colloids absorb and scatter the laser light, which can be seen by the outline of the sample holder. After the aggregation of colloids takes place, the colloidal solution gets transparent for the laser light. Here, huge volume fractions arise, where almost no colloid is present and the light is able to pass (Fig. 3).

### Colloidal disintegration

**Figure 4:**
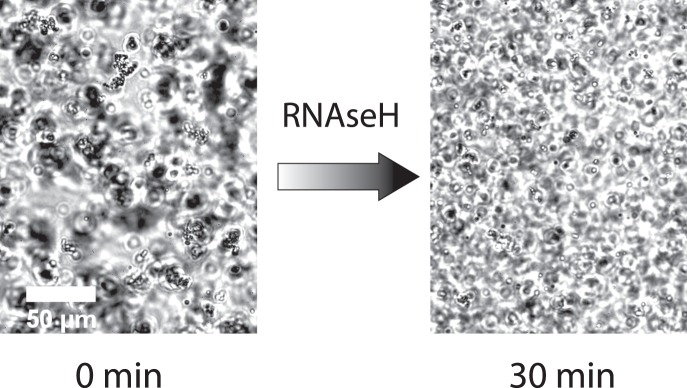
Colloidal disintegration. The colloidal aggregation is induced by the addition of 100 nM RNA-linker strands. The complete disintegration of the colloidal structures was realised using a *RNaseH* concentration of 0.1 U/μl. The cluster size decreased from 103 a.U. to 24 a.U. within 30 min.

In a next step, we test the enzymatic disintegration of the colloidal clusters by fragmenting the RNA-linker strands using the enzyme *RNaseH*. Pre-built RNA-linker (100 nM) were added to a colloidal solution containing 0.1 U/μl of *RNaseH*. After the structure formation was completed (20 min), the disassembly of the structures was observed within 30 min (Fig. 4). Also colloidal gels formed by the enzymatic RNA-linker production of the *T7-polymerase* were disintegrated in this time period (S6).

### Autonomous transient structure formation

Finally, we aimed to create an autonomously aggregating colloidal system, which exhibits a transient cluster aggregation without external intervention. Therefore, we had to identify the underlying kinetic parameters of the enzymatic reaction precisely. We assume that the system consists of a linear production and a linear degradation reaction. The speed of production depends on the concentration of *T7-Polymerase* (T7), the double-stranded DNA template (Temp) and the substrate NTP, while the degradation depends on the concentration of *RNaseH* and the total amount of RNA. By that, the enzymatic reaction can be described by Michaelis-Menten-Kinetics.

1 $$\frac{d[RNA]}{dt}=k1\ast T7\ast Temp\ast NTP-k2\ast RNaseH\ast [RNA]$$

**Figure 5:**
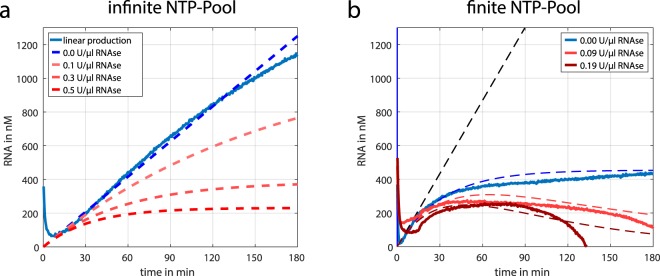
Transient RNA production. (**a**) The RNA production and degradation are linear reactions. In the absence of degradation, a linear production is achieved (blue curve). Adding a degradation term, results in a steady state were production and degradation equilibrates. The dashed lines represent the calculations obtained from eq. 1. (**b**) To realise a transient behaviour with linear equations, fuel (NTP) consumption is necessary to reduce the production rate over time. The blue line shows the RNA production with a limited reservoir of NTP. Limited production and an additional degradation can be seen by the red lines. Here, transient behaviour can be observed, which can be controlled by varying the *RNaseH* concentration. The corresponding dashed lines represent the calculations obtained from eq. 2, which takes the fuel consumption into account. The black dashed line depicts the pure production without fuel consumption (eq. 1) and fits well with the production curve in the first 10 minutes.

With *k1* and *k2* the rates of production and degradation, respectively. The rate of production was obtained experimentally from fluorescence measurements in the absence of *RNaseH* (S3). Here, the *T7* and *NTP* concentrations were varied separately while the template concentration was kept constant in all experiments. Figure 5a depicts the time dependence of the RNA concentration for a fixed RNA production speed and different *RNaseH* concentration (0 to 0.5 U/μl) according to eq. 1. As expected, the presence of *RNaseH* does lead to an effectively slower production rate, which equilibrates to a steady state, but does not show transient behaviour. In this setup, an autonomous transient RNA “pulse” can only be realised by the depletion of the NTP pool. Here, the consumption of the substrate reduces the production rate continuously. This is achieved, once the *T7* enzyme concentration is sufficiently higher than the substrate concentration. Experiments were performed to identify the optimal relation between *T7 polymerase* and NTP concentration, which shows the reduction of RNA production (Fig. 5b, blue curve) without *RNaseH*. The comparison of the measurements and the simulations obtained from eq. 1 (black dotted line) reveals the influence of the NTP consumption. Thus, eq. 1 can be expanded with the following NTP consumption term.

2 $$\frac{d[NTP]}{dt}=-k3\ast [NTP]\ast T7\ast Temp$$

Here, the rate *k3* represents the amount of nucleotides used to build one RNA strand. This set of equations nicely describes the observed behaviour. The addition of degradation reactions to such a limited RNA production leads then to a transient behaviour which can be controlled by the concentration of *RNaseH* (Fig. 5b, red curve). However, for higher *RNaseH* concentrations, a deviation compared to the simulations can be seen. This is possibly caused by interactions of small and less fluorescent RNA fragments or monomers, which result from the degradation process.

**Figure 6:**
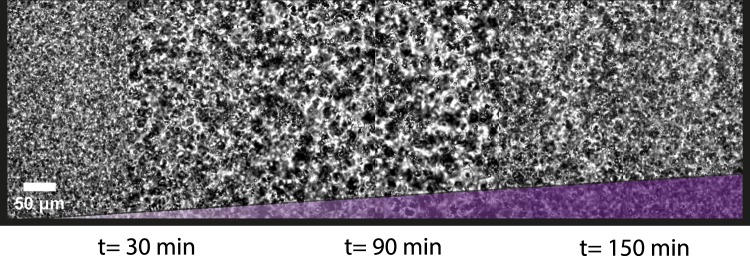
Transient colloidal structure formation. Colloidal structure formation is induced using the enzymatic setup of Fig. 5b (0.19 U/μl *RNaseH*). The structure formation starts after several minutes and reaches its peak after one hour of polymerisation. The aggregation is followed by a disintegration, which is completed in approximately two hours.

Subsequently, we coupled the colloidal system to the enzymatic reaction conditions of the transient RNA pulse (0.19 U/μl *RNaseH*). As a result, we were able to observe the transient aggregation of the colloids without external stimuli (Fig. 6). The aggregation is initiated from the very beginning and has a lifetime of around two hours. Thus, the colloidal aggregation matches very well with the corresponding RNA concentration profile (Fig. 5b).

#### Tuning the Transient structure formation

**Figure 7:**
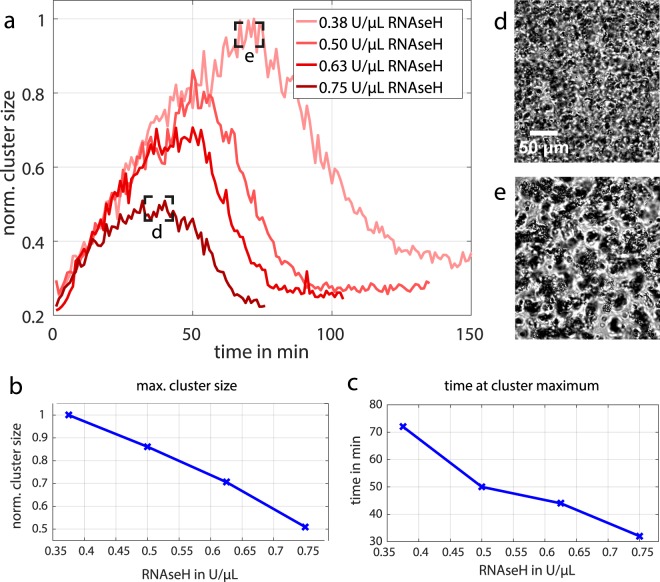
Controlling the colloidal structure formation. (**a**) The transient structure formation is controlled in terms of lifetime and cluster size by varying the enzymatic reactions. Here, the RNA production was kept constant, while the degradation was increased. (**b**) The maximal cluster size decreases with increasing *RNaseH* concentration. (**c**) The time at which the maximal cluster size occurs shortens with increasing *RNaseH* concentration. (**d**,**e**) Bright field images of the maximal cluster size for the shortest and the longest pulse, respectively.

The direct dependency of the colloidal structure formation and the enzymatic reaction enables a further temporal control of the transient aggregation. To this end, we used the obtained parameter dependencies of the RNA pulses (Fig. 5b) and increased the *RNaseH* concentration, while the production rate and substrate concentration were kept constant. Image analysis was performed to quantify the lifetime and the average aggregation size of the structure formation over time (Fig. 7a). As expected, the lifetime of the colloidal aggregates can be tuned and increases successively (70 to 150 min) with decreasing *RNaseH* concentration. The total cluster size is directly associated to the lifetime, because of the longer aggregation time and increases from approximately 20 to 60 μm in diameter (Fig. 7b,d,e). It is noticeable, that the resulting colloidal aggregation pulses behave similar in the first 30 min. Here, the kinetics are dominated by the RNA production rate. In contrast, the initiation of degradation is delayed by the lower *RNaseH* concentrations (Fig. 7c). Further experiments were performed keeping the enzymes constant while varying the NTP pool (S7). Also, these samples showed transient clustering behaviour, but did not facilitate the sensitive tuning of lifetime and cluster size.

#### Tuning the transient structure formation by competing hybridisation reactions

**Figure 8:**
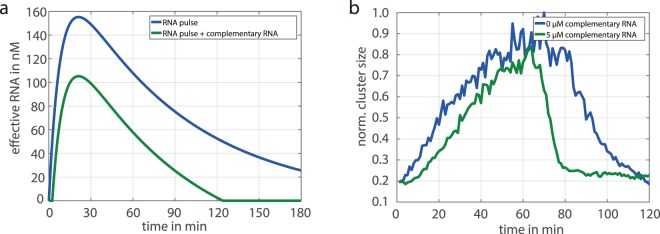
Controlling transient colloidal structure formation by hierarchical hybridisation reactions. (**a**) The transient RNA production (blue line) has a fast initial phase and is followed by a relative slow degradation. Adding a complementary RNA to the system (here 50 nM) reduces the unbound RNA, which is potentially able to link colloids (green line) (**b**). Comparison of the transient structure formation with and without complementary RNA. The complementary RNA-linker acts like an aggregation threshold and delays the aggregation and accelerates the disintegration.

Here, we harness the selectivity of DNA/RNA hybridisation to tune the colloidal aggregation using competing hybridisation reactions. RNA strands, which are fully complementary to the RNA-linker were added freely to the solution. The produced RNA-linkers first bind to the free complementary RNA strands because of the high affinity. The overlap of the RNA-linker to each DNA sequence of the beads is only half the length and has by that a lower affinity. Additionally, the RNA/RNA duplex is protected from degradation, because the *RNaseH* is only able to degrade RNA when it’s bound to DNA. Accordingly, more RNA-linker than free complementary RNA has to be produced, until the colloidal aggregation initiates. Thus, the added complementary RNA acts like an aggregation threshold. By using this threshold, the shape of the “effective” single-stranded RNA-linker can be tuned, here demonstrated for the transient RNA pulse (Fig. 8a). This setup was also coupled to the colloidal system (Fig. 8b). The colloidal clustering is delayed by around 10 minutes and the effective disintegration process is much faster. Just a few minutes are required and a very abrupt initiation can be observed. This can be explained by the properties of the underlying RNA pulse. It is characterised by a relative fast production and a subsequent slow degradation phase. Consequently, the threshold has just a small influence at the fast initial phase of RNA synthesis. The amount of effective RNA, which is necessary for the aggregation, is reached within a few minutes. However, the time delay until the effective RNA is below a critical concentration leading to disintegration is significant.

#### Spatial control of colloidal aggregation

**Figure 9:**
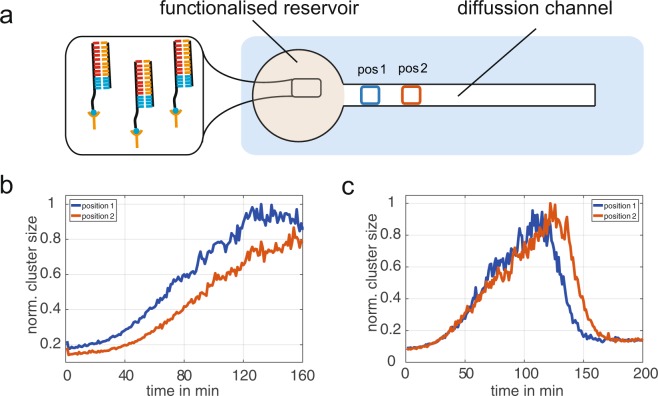
Propagation of aggregation front. (**a**) The reservoir of the diffusion chamber is functionalised with DNA Templates. By that, the RNA production is restricted locally, while the degradation takes place globally. The aggregation was analysed at different positions (distance 400 μm) in the diffusion channel. (**b**) RNA is produced locally at the reservoir and diffuses into the channel, resulting in an aggregation front, which propagates into the channel. (**c**) Using the enzymatic conditions of a transient RNA pulse leads to an aggregation, which starts almost simultaneously for both positions. Here, the disintegration starts at position 1 and propagates along the channel.

For the realisation of a spatial control of the structure formation process, we constructed a microfluidic chamber, in which the template strands were immobilized to confined areas on a cover glass in order to restrict the RNA production locally (Fig. 9a). As a consequence, the propagation of a colloidal aggregation front can be detected after the initiation of RNA polymerisation (Fig. 9b). The velocity of this aggregation is set by the diffusion of the newly synthesised RNA strands. At relatively high production rates, a homogenous aggregation near the production reservoir occurs in the field of view, indicating that the RNA gradient and the associated diffusion is high compared to the aggregation time. Applying the above characterised conditions of a transient RNA pulse to this diffusion chamber leads to an aggregation, which is followed by a disintegration front. It is noticeable that the disintegration front is traveling away from the production source (Fig. 9c). In general, a local produced RNA is diffusing into the channel, while the degradation takes place globally. Therefore, it is expected that the RNA concentration is always decreasing with respect to the RNA source. Consequently, the aggregation should propagate into the channel followed by a degradation in the backwards direction in contrast to the observed forward direction. Simple simulations show under all reasonable parameters the backwards RNA degradation leading to the backwards colloidal disintegration (S8a,c). Thus, a direct transfer of RNA concentration to colloidal aggregation is not sufficient. Additional control experiments were performed, demonstrating that colloids have a reduced affinity after aggregation and disintegration (S9). This can be explained, by the operation of *RNaseH*, which is just able to degrade RNA when it’s bound to DNA. It is important to realize, that most of the RNA-linkers do not bind two colloids together but decorate the free surfaces of the colloids. Once these complementary parts of the RNA-linkers are degraded, the remaining RNA is free to bind to the colloidal DNA again, effectively blocking remaining full length linker strands from bridging between two types of colloids. Computing the equilibration of all competing DNA/RNA hybridisation reactions confirm that the temporary by-products result in a reduction of the colloidal DNA – linker complex (S10). Consequently, the colloids have a “history” of aggregation, resulting in a temporary reduced affinity. Expanding the simulation with this reduced binding shows the forward propagation of a colloidal disintegration front, as observed in the experiments (S8b). The aggregation is moving through the channel and is followed by a disintegration front, resulting in a “traveling wave” of aggregation.

The presented approach allows the dynamic control of colloidal structure formation by enzymatic reactions, based on the well-established biochemical toolbox readily available for the manipulation of DNA molecules. Here, the fine control of colloidal interactions allows the tuning of the colloidal structure formation in terms of lifetime and total cluster size. Additionally, the hierarchical organisation of the reactions are a promising pathway to extend the system to a multi-species system and enable hierarchical guided assembly. It is straight forward to use the enzymatic setup for colloidal systems with different DNA sequences. The length of the DNA as well as the density on the colloids can be varied and result in different strengths of the colloidal interactions. Consequently, the approach is also accessible for colloidal systems with different sizes and materials. As the material properties of the colloidal aggregates are mainly defined by the properties of the single building blocks, many different applications can be addressed, such as biosensors by using plasmonic gold nanoparticles^36,37^. Especially the transient control of colloidal organisation may proof to be useful to facilitate the development of active and autonomously acting materials.

## Methods

### DNA coating and sample preparation

Fluorescent, streptavidin coated microspheres (Polysciences) with a diameter of 1 μm were functionalized with 1.7 μM of biotinylated DNA strands (biomers) for at least 6 hours and washed by centrifugation (6000 rcf) afterwards. The sequences of this constructs are: Docking A: 5′-CCACACCAACCAAC-3′- biotin and Docking B: biotin -5′-ACTTACTATATAAC-3′. The sequence of the dsDNA Template is: 5′-CACCCACCCACACCAACCAACAAAACTTACTATATAACCCCTATAGTGAGTCGTATTAG-3′ and the complementary part: 5′-CTAATACGACTCACTATAGGGGTTATATAGTAAGTTTTGTTGGTTGGTGTGGGTGGGTG-3′ – (biotin) with an optional biotin modification for the functionalisation of the coverslides used in the diffusion experiments. The linker $\overline{AB}$ sequence is: 5′-GTTGGTTGGTGTGGGTGGGTGTTTGTTATATAGTAAGT-3′ and the complementary RNA used in the threshold experiments is: 5′- ACUUACUAUAUAACAAACACCCACCCACACCAACCAAC-3′. All proteins (ThermoFisher Scientific) were used undiluted at their stock solution: *RNaseH* (5 U/μL) and T7 RNA Polymerase (200 U/μL). The colloidal aggregation experiments were performed with a total concentration of 30 mM MgCl_2_ for the optimal activity of T7 RNA Polymerase and 450 mM sucrose to avoid the sedimentation of the colloids. The colloids were used at 0.15 vol.-% and 5 mg/ml BSA was used to avoid the stickiness of the glass surface. The working temperature was T = 37 °C for all experiments. Therefore, an IBIDI heating-chamber and a copper objective warmer was used. For the diffusion experiments, parafilm was cutted in the shape shown in Fig. 9a using a laser cutter. The reservoir was functionalised with biotinylated templates using BSA-Biotin and streptavidin.

### Microscopy and fluorescence spectroscopy

Bright-field optical images and videos were obtained using a Leica DMI6000B and an HCX PL APO 40x/1.25–0.75 oil CS objective. For the confocal microscopy, a Leica TCS SP5 was used and a HCX PL APO 63x/1.40–0.60 oil CS objective. The fluorescence measurements were performed using a Jasco Spectrofluorometer FP-8500 and the DNA/RNA intercalator SyBr GreenII. The fluorescence signal (a.U.) is transferred to the total RNA concentration. To this end, the minimum of the signal during the RNA production was determined and computed to be the DNA template concentration in solution, because at this time point no RNA was produced so far. This calibration allows a direct conversion of the fluorescence signal to the DNA concentration. In control measurements we confirmed the linear relation between fluorescence signal and DNA concentration up to 10 μM.

### Data analysis and computer simulation

The average cluster size of the colloidal aggregates is determined by thresholding the bright field images to create a binary image. The obtained masks of the colloidal aggregates were analysed in terms of size and total amount. The simulations were performed in rectangular matrices using periodic boundary conditions for the colloids and repulsive boundary conditions for the RNA. Differential equation (eq. 2) were used to calculate the RNA production and degradation and the diffusion was simulated by using a gaussian-filter function.

## Supplementary information

Supplementary Informations

## References

[1] Rueb CJ, Zukoski CF (1997). Viscoelastic properties of colloidal gels. Journal of Rheology.

[2] Shih, W.-H. *et al*. Mechanical Properties of Colloidal Gels. *MRS Proc*. **155** (1989).

[3] Zhou Y (2018). Unusual multiscale mechanics of biomimetic nanoparticle hydrogels. Nature communications.

[4] Kamp SW, Kilfoil ML (2009). Universal behaviour in the mechanical properties of weakly aggregated colloidal particles. Soft matter.

[5] Park J-G (2014). Full-spectrum photonic pigments with non-iridescent structural colors through colloidal assembly. Angew. Chem. Int. Ed..

[6] Fan JA (2010). Self-Assembled Plasmonic Nanoparticle Clusters. Science.

[7] Dehne H, Hecht FM, Bausch AR (2017). The mechanical properties of polymer-colloid hybrid hydrogels. Soft matter.

[8] Wang Y, Jenkins IC, McGinley JT, Sinno T, Crocker JC (2017). Colloidal crystals with diamond symmetry at optical lengthscales. Nature communications.

[9] Khlebtsov NG (2000). Light Absorption by the Clusters of Colloidal Gold and Silver Particles Formed During Slow and Fast Aggregation. Colloidal. Journal.

[10] Grünwald M, Geissler PL (2014). Patterns without patches. Hierarchical self-assembly of complex structures from simple building blocks. ACS nano.

[11] Liang Y, Hilal N, Langston P, Starov V (2007). Interaction forces between colloidal particles in liquid. Theory and experiment. Advances in colloid and interface science.

[12] Meakin P (1983). Formation of Fractal Clusters and Networks by Irreversible Diffusion-Limited Aggregation. Physical Review Letters.

[13] Masuda Y, Itoh T, Koumoto K (2005). Self-Assembly Patterning of Silica Colloidal Crystals. Langmuir.

[14] Chen Q, Bae SC, Granick S (2011). Directed self-assembly of a colloidal kagome lattice. Nature.

[15] Lu PJ (2008). Gelation of particles with short-range attraction. Nature.

[16] Mani E, Lechner W, Kegel WK, Bolhuis PG (2014). Equilibrium and non-equilibrium cluster phases in colloids with competing interactions. Soft matter.

[17] Dinsmore AD, Crocker JC, Yodh AG (1998). Self-assembly of colloidal crystals. Current Opinion in Colloid & Interface Science.

[18] Wang Y (2015). Crystallization of DNA-coated colloids. Nature communications.

[19] Angioletti-Uberti, S., Varilly, P., Mognetti, B. M. & Frenkel, D. Mobile linkers on DNA-coated colloids. Valency without patches. *Phys. Rev. Lett*. **113** (2014). 10.1103/PhysRevLett.113.128303 25279648

[20] McGinley JT, Wang Y, Jenkins IC, Sinno T, Crocker JC (2015). Crystal-Templated Colloidal Clusters Exhibit Directional DNA Interactions. ACS nano.

[21] Park SY (2008). DNA-programmable nanoparticle crystallization. Nature.

[22] Cigler P, Lytton-Jean AKR, Anderson DG, Finn MG, Park SY (2010). DNA-controlled assembly of a NaTl lattice structure from gold nanoparticles and protein nanoparticles. Nature materials.

[23] Nykypanchuk D, Maye MM, van der Lelie D, Gang O (2008). DNA-guided crystallization of colloidal nanoparticles. Nature.

[24] Weitz DA, Lin MY (1986). Dynamic Scaling of Cluster-Mass Distributions in Kinetic Colloid Aggregation. Physical Review Letters.

[25] Hecht FM, Bausch AR (2016). Kinetically guided colloidal structure formation. Proceedings of the National Academy of Sciences of the United States of America.

[26] Zaccarelli E (2007). Colloidal Gels. Equilibrium and Non-Equilibrium Routes. J. Phys.: Condens. Matter.

[27] Weitz DA, Huang JS, Lin MY, Sung J (1984). . Dynamics of Diffusion-Limited Kinetic Aggregation. Physical Review Letters.

[28] Di Michele L (2013). Multistep kinetic self-assembly of DNA-coated colloids. Nature communications.

[29] von Maltzahn G (2007). Nanoparticle Self-Assembly Directed by Antagonistic Kinase and Phosphatase Activities. Adv. Mater..

[30] Dreyfus R (2010). Aggregation-disaggregation transition of DNA-coated colloids. Experiments and theory. Physical review. E, Statistical, nonlinear, and soft matter physics.

[31] Angioletti-Uberti S, Mognetti BM, Frenkel D (2012). Re-entrant melting as a design principle for DNA-coated colloids. Nature Mater.

[32] van Ravensteijn BGP, Hendriksen WE, Eelkema R, van Esch JH, Kegel WK (2017). Fuel-Mediated Transient Clustering of Colloidal Building Blocks. Journal of the American Chemical Society.

[33] Boekhoven J (2015). Transient assembly of active materials fueled by a chemical interaction. Science.

[34] Park N, Um SH, Funabashi H, Xu J, Luo D (2009). A cell-free protein-producing gel. Nature materials.

[35] Roh YH, Ruiz RCH, Peng S, Lee JB, Luo D (2011). Engineering DNA-based functional materials. Chemical Society Reviews.

[36] Buchmann B, Hecht FM, Pernpeintner C, Lohmueller T, Bausch AR (2017). Controlling Non-Equilibrium Structure Formation on the Nanoscale. Chemphyschem: a European journal of chemical physics and physical chemistry.

[37] Wang C, Chen Y, Wang T, Ma Z, Su Z (2007). Biorecognition-Driven Self-Assembly of Gold Nanorods. A Rapid and Sensitive Approach toward Antibody Sensing. Chem. Mater..

