# Supplementary material for: Transient self-organisation of DNA coated colloids directed by enzymatic reactions
Source: Sci Rep. 2019 May 14;9:7350. doi: 10.1038/s41598-019-43720-7 (PMC6517385; doi:10.1038/s41598-019-43720-7)
Supplement: Supplementary file 1 — Supplementary Informations [file 41598_2019_43720_MOESM1_ESM.pdf]

Supplementary Information

Transient self-organisation of DNA coated colloids directed by enzymatic reactions

Dehne, H., Reitenbach, A. and A.R. Bausch

(S1) Reconstruction of the colloidal aggregation by confocal imaging

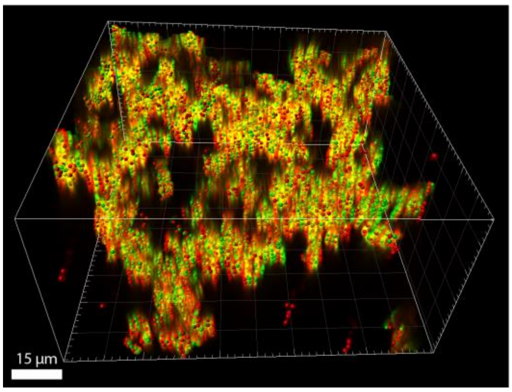

The colloidal aggregation was observed after two hours of RNA-linker polymerisation using confocal microscopy. The reconstructions of the colloidal gel was performed to proof the functionality of the RNA-linker to bind two different kinds of colloidal docking DNA, which results in alternating binding of red and green colloids.

(S2) Linear fluorescence and DNA dependency

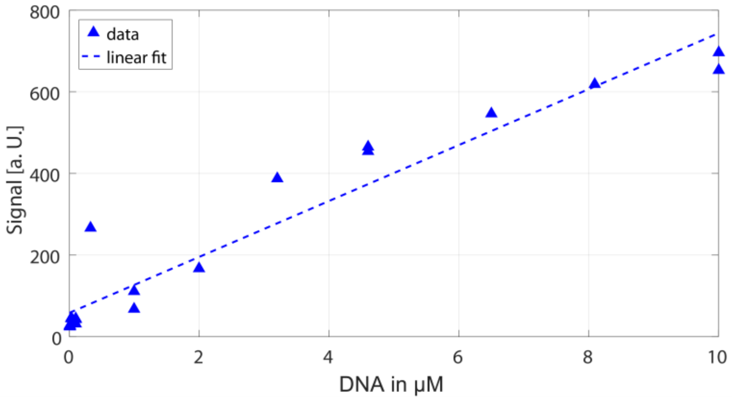

The dye *SyGr II* obeys a linear dependency of fluorescence and DNA concentrations up to 10 µM without saturation. By that, the fluorescence signal can be transferred into RNA concentration. For the performed polymerisation experiments, the fluorescence of the used templates as well as the absorption and auto fluorescence of enzymes has to be taken into account.

(S3) Linear dependency between RNA production rate and NTP/T7 concentration

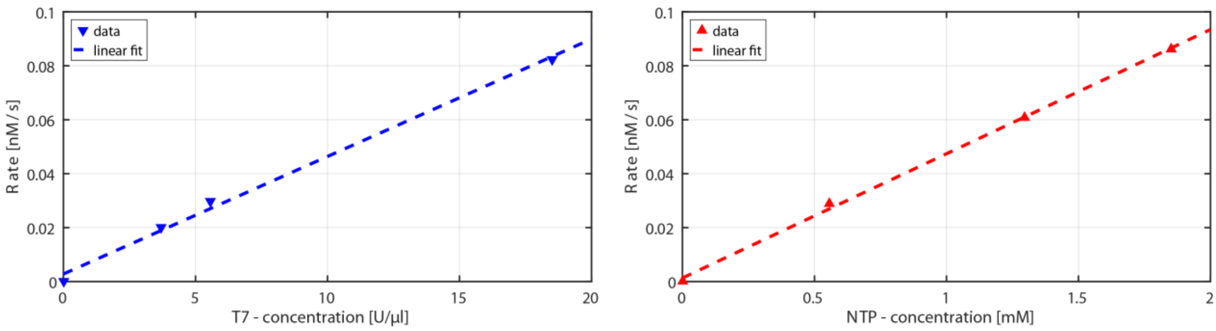

A linear dependency between RNA production rate and NTP and *T7* concentration can be observed. Thus, it is possible to calculate the RNA production rates for arbitrary combinations of enzymes:

(S4) Colloidal aggregation over time for different RNA production rates

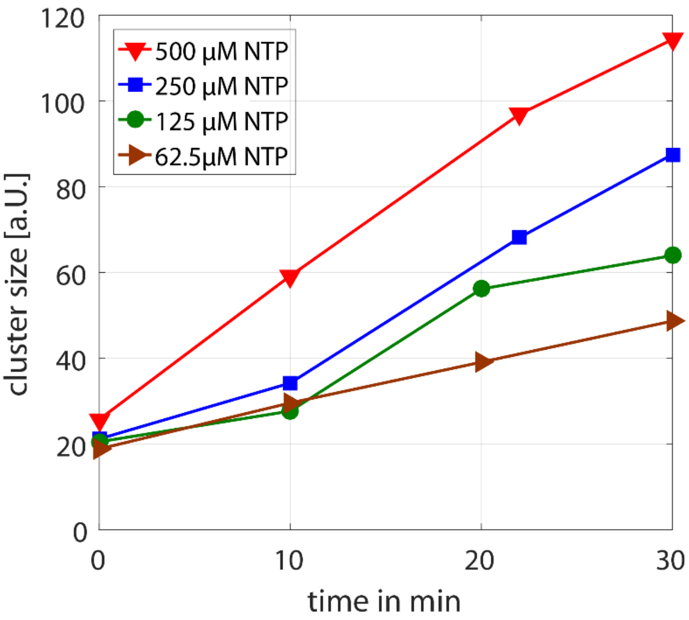

The speed of aggregation is controlled by tuning the speed of RNA-linker polymerisation. The production rate is adjusted by varying the NTP concentration and can be transferred to the speed of aggregation.

(S5) Polymerisation of RNA-Linker with different colloids

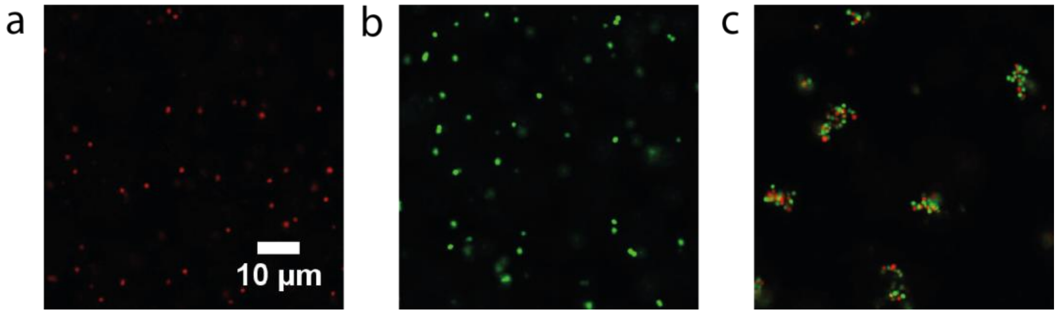

The polymerisation of RNA linker was performed in the presence of colloids functionalised with docking A (**a**), docking B (**b**) and the combination A+B (**c**). Only the combination of both docking strands form clusters. By that it is possible to proof the functionality of the produced RNA linker and the selectivity of the DNA hybridisation.

(S6) Colloidal disintegration by *RNAseH*

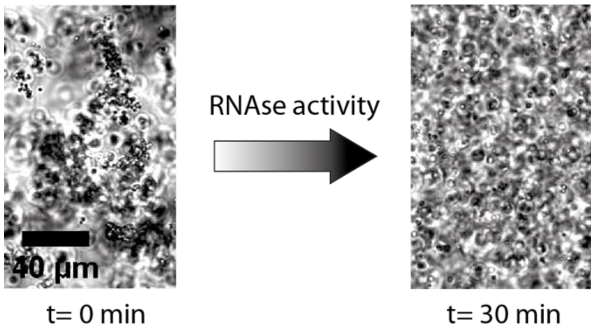

The functionality of the *RNAseH* to disintegrate the colloidal aggregation was observed after the enzymatic induced aggregation was completed. A fully disintegration of the colloids could be monitored within 30 min. In detail, the RNA is first produced by the T7 polymerase with a NTP concentration, which is depleted after two hours of polymerisation. The *RNAseH* is added after this process. As pipetting causes shear forces and fragmentation of the colloidal structures, we cooled down the sample to 4°C. RNA is not degraded at this temperature, due to the temperature sensitivity of the *RNAseH*. In contrast, the colloids are able to aggregate again, as the previously produced RNA is still present in solution. After two hours of colloidal aggregation, the sample was heated back to working temperature (37°C) to activate the *RNAseH*. A complete disintegration of the colloidal aggregates can be observed within 30 min. By that it was possible to proof the functionality of the used enzymes to induce aggregation and disintegration of the colloids, by producing and degrading RNA-linker.

(S7) Transient aggregation by varying the NTP concentrations

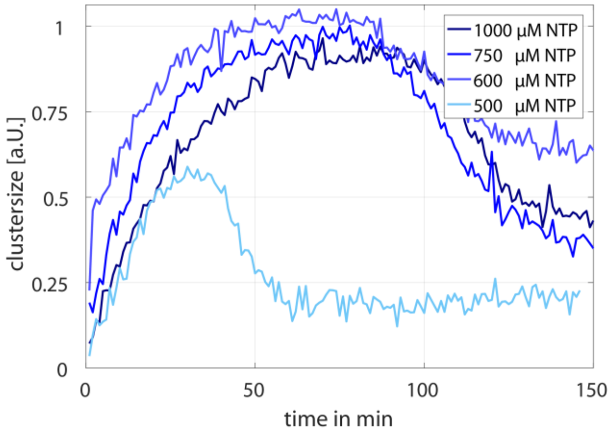

Transient aggregation was realised by keeping the enzyme concentrations constant and just vary the NTP concentration. The samples with 1000, 750 and 600 µM NTP show similar behaviour. Just the sample with 500 µM NTP differs.

(S8) Simulation of the propagating aggregation and disintegration front

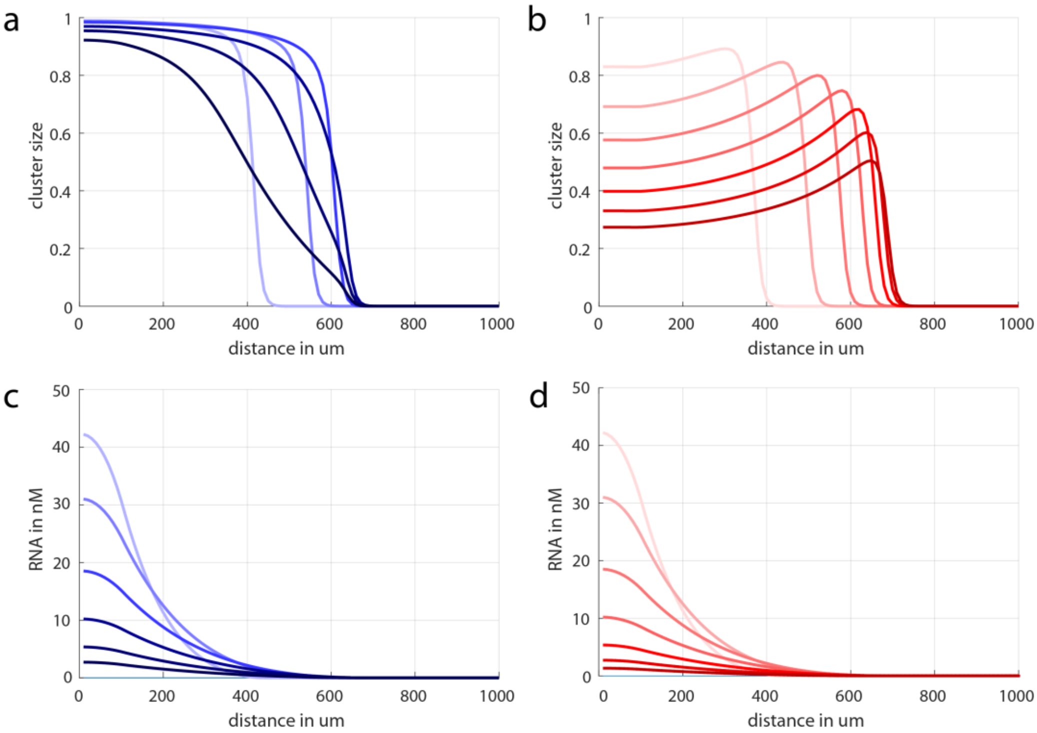

Simulations were performed to analyse the directed propagation of the colloidal aggregation. Here, the increase of the colour strength represents the increase of time. Locally produced RNA diffuses into the channel and is degraded globally. (**a**) The direct transfer of RNA concentration to the colloidal aggregation leads to a front propagation and backwards disintegration. (**b**) Under consideration of a reduced binding of disintegrated beads, you can see the propagation of an aggregation and disintegration front in the forward direction. (**c, d**) The corresponding RNA concentrations are similar for both cases.

(S9) Reduced colloidal affinity after disintegration

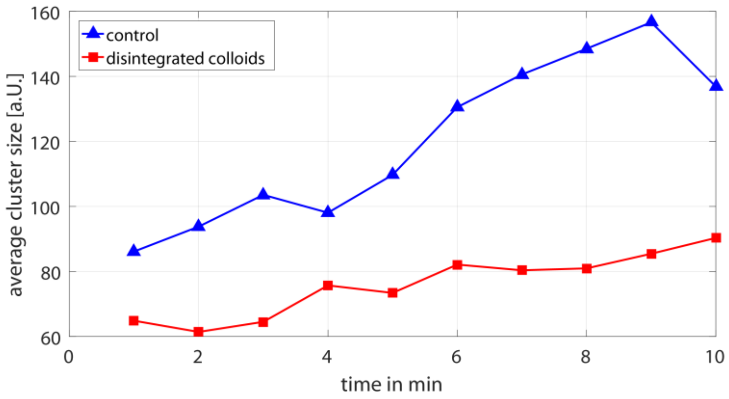

Here, the affinity of “new” colloids and already disintegrated colloids is compared. Therefore, colloids were incubated with *RNAseH* and 10 µM RNA linker. After two hours the aggregation and disintegration is completed. In a next step, the aggregation is induced by the addition of DNA Linker. A reduced affinity of the disintegrated colloids can be observed.

(S10) DNA Hybridisation of reduced linker

The influence of partial degraded RNA Linker was verified using *NUPACK* (http://www.nupack.org/). At 1 µM of Docking A, B and the RNA-linker, 920nM of A-B-Linker complexes are formed at working temperature. After adding 1 µM of reduced Linker, the A-B-Linker complex concentration is reduced to 460nM.
